# Adaptive design: adaptation and adoption of patient safety practices in daily routines, a multi-site study

**DOI:** 10.1186/s12913-020-05306-2

**Published:** 2020-05-14

**Authors:** Connie Dekker - van Doorn, Linda Wauben, Jeroen van Wijngaarden, Johan Lange, Robbert Huijsman

**Affiliations:** 1 Rotterdam University of Applied Sciences, Research Centre Innovations in Care, Rochussenstraat 198, 3015 EK Rotterdam, The Netherlands; 2 Erasmus University Medical Center, Department of Surgery, P.O. Box 2040, 3000 CA Rotterdam, The Netherlands; 3 Delft University of Technology, Department of BioMechanical Engineering, Faculty of Mechanical Engineering, Mekelweg 2, 2628 CD Delft, The Netherlands; 4 Erasmus University Rotterdam, Erasmus School of Health Policy & Management, P.O. Box 738, 3000 DR Rotterdam, The Netherlands

**Keywords:** Structured bottom-up implementation approach, Engaging professionals, Design, Learn

## Abstract

**Background:**

Most interventions to improve patient safety (Patient Safety Practices (PSPs)), are introduced without engaging front-line professionals. Administrative staff, managers and sometimes a few professionals, representing only one or two disciplines, decide what to change and how. Consequently, PSPs are not fully adapted to the professionals’ needs or to the local context and as a result, adoption is low. To support adoption, two theoretical concepts, Participatory Design and Experiential Learning were combined in a new model: Adaptive Design. The aim was to explore whether Adaptive Design supports adaptation and adoption of PSPs by engaging all professionals and creating time to (re) design, reflect and learn as a team. The Time Out Procedure (TOP) and Debriefing (*plus*) for improving patient safety in the operating theatre (OT) was used as PSP.

**Methods:**

Qualitative exploratory multi-site study using participatory action research as a research design. The implementation process consisted of four phases: 1) start-up: providing information by presentations and team meetings, 2) pilot: testing the prototype with 100 surgical procedures, 3) small scale implementation: with one or two surgical disciplines, 4) implementation hospital-wide: including all surgical disciplines. In iterations, teams (re) designed, tested, evaluated, and if necessary adapted TOP*plus*. Gradually all professionals were included. Adaptations in content, process and layout of TOP*plus* were measured following each iteration. Adoption was monitored until final implementation in every hospital’s OT.

**Results:**

10 Dutch hospitals participated**.** Adaptations varied per hospital, but all hospitals adapted both procedures. Adaptations concerned the content, process and layout of TOP*plus*. Both procedures were adopted in all OTs, but user participation and time to include all users varied between hospitals. Ultimately all users were actively involved and TOP*plus* was implemented in all OTs.

**Conclusions:**

Engaging all professionals in a structured bottom-up implementation approach with a focus on learning, improves adaptation and adoption of a PSP. As a result, all 10 participating hospitals implemented TOP*plus* with all surgical disciplines in all OTs. Adaptive Design gives professionals the opportunity to adapt the PSP to their own needs and their specific local context. All hospitals adapted TOP*plus*, but without compromising the essential features for its effectiveness.

## Background

Interventions to improve patient safety are complex and hard to implement without the support of front-line healthcare professionals (Pearson et al., 2009). These interventions, also called Patient Safety Practices (PSPs) aim to prevent and mitigate unintended consequences of healthcare delivery and improve patient safety [1]. Most PSPs are the result of new guidelines, rules or regulations developed at a national or international level, and may include a wide variety of interventions, such as the introduction of protocols, process redesign, or team training. However, in most healthcare organizations only a mono-disciplinary, and limited group of healthcare professionals or administrative staff is involved in the introduction of PSPs and in what to change and how. Implementation in local practices are often top-down and mandatory without the necessary adaptations to address the needs of front-line professionals in that particular local context [2–4]. As a result, PSPs are not regarded as meaningful, and not at all or only partially adopted as a daily work routine [5, 6].

Most PSPs are complex interventions and involve adaptations in clinical processes and individual work routines, but also, to secure adoption, involve changes in personal or team behaviour [1]. The majority of patients admitted to hospital is complex and treated by multidisciplinary teams of professionals, supported by administrative personnel and sophisticated technology [7]. Consequently, initiatives to improve healthcare delivery processes demand a high degree of cooperation and collaboration among and between teams [2]. As learning new behaviours is often hampered by existing behaviours, implementation strategies should aim at learning new behaviour, but aim also at unlearning old routines [8]. Therefore, successful introduction of PSPs requires active engagement of all front-line staff [9–11].

In existing theoretical concepts for implementation, authors emphasize the necessity of engaging professionals to improve adoption and discuss the impact of the local organisational context, which might necessitate adaptation of PSPs [2, 12]. The authors discuss facilitators, and barriers for implementation, emphasize a system approach, and present steps for implementation [13, 14]. However, most articles provide little information on how to choose the right strategies, and how and when to include the healthcare professionals from the clinical units. Moreover, there is also a danger that by adapting PSPs, essential features are lost and the intervention becomes less effective. According to T Greenhalgh, G Robert, F Macfarlane, P Bate and O Kyriakidou [15] a more theory-driven, process-oriented, and participatory approach is needed. R Foy, J Ovretveit, PG Shekelle, PJ Pronovost, SL Taylor, S Dy, S Hempel, KM McDonald, LV Rubenstein and RM Wachter [1] recommended combining implementation strategies from different theoretical domains, e.g. healthcare logistics and behavioural sciences to address the different kind of problems that might occur in the process of implementation.

In a preliminary study in 5 hospitals [16], we developed and tested a new approach for implementation of a team procedure to increase face-to-face communication in the operating theatre (OT). A time out procedure to exchange critical information as a team pre-operatively, and a debriefing to discuss possible complications and consequences post-operatively improve patient safety. To engage professionals in the design and implementation process we used Participatory Design (PD), a theoretical concept from Industrial Design Engineering, as implementation strategy. PD is a user-centred design strategy to support adaptation of the product or process and improve actual usage, without compromising essential design features. With PD a small group of potential end-users participates in all phases of the design and redesign process in a structured, efficient and safe way, which in turn leads to an improved user-oriented design, adapted to the local context [9, 17]. However, the implementation process in our preliminary study showed that: 1) adaptation of both time out procedure and debriefing between hospitals, was far more diverse than expected, and 2), teams needed more time and moments to experiment, reflect and learn as a team to adapt both time out procedure and debriefing, and adopt it as daily routine procedures. It was evident that for full implementation, more time was needed to support the collective process of team learning, and to include all professionals involved in that process.

Our proposition for the present study was that successful implementation needs an integrated approach to (re) design, to experiment and to learn as a team. To expand the design process to a team learning process for *all* professionals involved in the present study, Participatory Design (PD) was combined with Experiential Learning (EL). In this study the focus is on (re) design and implementation of two procedures: 1) a Time Out Procedure (TOP), a double-check that takes place just before incision, the first step in the surgical procedure in OT and 2) a Debriefing (*plus*) performed just before skin closure [16, 18, 19]. TOP*plus* is a communication tool to support and improve communication and teamwork between OT team members by *discussing* and *checking* important items preoperatively (in comparison to traditional checklists which mainly focus on checking). At the start of our project, checklists were not mandatory in the Netherlands, making TOP*plus* new.

At first, we focused on engaging all professionals in the design and implementation process, as at the start of the TOP *plus* project professionals were reluctant to involve patients, partly because of the patient’s pre-medication and their answers not always being reliable.

This study focuses on the introduction of TOP*plus* for OT teams in 10 Dutch hospitals.

The aim of this study was to explore whether a bottom-up integrated approach combining PD and EL supports *adaptation* and *adoption* of TOP*plus*, without compromising essential design features. To improve effectiveness, TOP*plus* might need adaptations to fit the needs of the professionals and the care processes in specific local environments. Adoption by all professionals is required for the procedure to be successful and reduce preventable complications and unnecessary readmissions.

### Theoretical background

In this study, the two theoretical concepts, Participatory Design and Experiential Learning are integrated into a new model for implementation: Adaptive Design.

### Participatory Design (PD) - Structures the Design and Implementation process

PD is a design-oriented research methodology, which is highly iterative and supports systematic input in different design cycles. PD was developed to involve end-users actively in the design and decision-making processes [9, 17]. The PD process comprises four primary steps: Design; Test; Evaluate; and Redesign in which a small group of experts and end-users develops a prototype. Then, another group of end-users is invited to test the prototype and provide feedback to improve the design and the usability of the product. To prevent the loss of essential features or criteria that are critical for the product or process to be effective, experts safeguard these basic criteria. For usage of TOP*plus* in OT, the basic criteria included e.g. questions to prevent wrong side, wrong person and wrong surgical intervention and a question about possible allergies. Research has shown that PD leads to more a user-oriented, context specific design and improves actual usage [9]. Therefore*,* we assume that applying PD principles structures the design, redesign and implementation process to adapt TOP*plus* without sacrificing essential criteria.

### Experiential Learning (EL) - Structures the Learning Process.

Including all professionals in the learning process requires special attention for individual and team learning during implementation of PSPs [20, 21]. To overcome resistance and improve adoption rates, extra time is needed to learn from experience and adapt PSPs to the local context [16]. EL emphasizes the value of successive learning cycles of end-users, where knowledge can be created and recreated through experience and reflection at individual and at team level. In these learning cycles, all professionals can be involved in the design and implementation process of the PSP, and make it effective in their own local work environment [22].

EL is defined as *‘The process whereby knowledge is created through the transformation of experience. Knowledge results from the combination of grasping and transforming experience’* DA Kolb [22] (p41). In contrast to didactic learning, with EL knowledge is created and re-created through experience e.g. small group discussions about daily practice, or interprofessional learning experiences, such as simulations. EL includes four steps: Learn; Experience; Reflect & Learn; and Act. The participating professionals actively reflect on their experiences and determine whether and how the PSP improves patient safety and how it affects the work environment [23]. Therefore, we assume that combining PD with EL principles improves the team learning process to support adaptation of TOP*plus* to the local context and adoption by all professionals involved.

### Adaptive Design.

Adaptive Design (AD) blends design and learning cycles in which designers and professionals learn as a team and redesign TOP*plus* in consecutive iterative design and learning cycles (Table 1). AD is a pro-active approach leading to a tentative end-product, which needs structural monitoring to adapt it to its users and context when necessary. Although AD and the plan–do–check–act cycle (PDCA) method are comparable on characteristics like being cyclic and focussing on small steps and close observations, PDCA is mostly reactive and leads to a new standard or final end-product.

**Table 1:** Adaptive Design: a model to improve adaptation and adoption of patient safety practices

	Process	Participatory Design (PD)	Experiential Learning (EL)	Adaptive Design (AD)
**Process characteristics**	Adaptation/ Adoption	Adoption	Adaptation	Adoption & Adaptation
	Orientation	Product	Process	Product & Process
	Design/ learning cycles	Structured & Iterative	Unstructured & iterative	Semi-structured & iterative
	Knowledge	Objective	Subjective Created & recreated	Objective & Subjective Created & recreated
	End-product	Final	Final (uncertain)	Provisional
**Design process**	Process Steps	Design Test Evaluate Redesign	Learn Experience Reflect & Learn Act	Design & Learn Test & Experience Evaluate, Reflect & Learn Redesign & Act
	User participation	Small, ad hoc end-user group	End-users randomly chosen from designated group	All end-users from designated group
	Influence	Limited	No pre-set limits	Adaptable within pre-set limits
	Acceptance	Early adoption	Adoption varies between end-users or user groups	Early adoption by small group, increasingly adopted by more users, eventually by all end-users
	Learning	Participating end-user group	Individuals & teams from designated users	All users from designated group: individual, team & organisation
	End-product	One for all end-users	Might be different between end-users (user groups)	Provisional per user group, subject to change over time

PD and EL reinforce each other by their iterative and user-centred approach, thus supporting adoption of the procedure by professionals and adaptation of the procedure to the specific local context. Adaptive Design creates the possibility to redesign and apply the procedure (act) within their context in iterative cycles. The pre-set limits are discussed and agreed upon beforehand with the teams, which will prevent discussions afterwards.

One of the main features of Adaptive Design is to include all professionals directly involved in the care process, including administrative and support staff as implementation of both procedures might lead to changes in administrative processes or IT support systems. Moreover, team discussions and decisions to adapt care processes, might also initiate consultations with other clinical departments or in the end lead to adaptations of organizational policies.

#### Participants

In each hospital, three core groups were actively involved in the design and implementation process:

1. the design expert team: a small team of designers and key-users representing all disciplines in OT who are well-informed about national and international rules and regulations;
2. the local expert team: a group of users representing all disciplines involved in surgical care (surgical, nursing, anaesthesiology, managerial, support staff) and familiar with the local context;
3. all OT team members: all healthcare providers from that local context, that are directly involved in surgical care.

The design expert team consisted of six team members: one surgeon, one anesthesiologist, one OT-nurse, one OT-manager and two researchers (one with a background in Industrial Design Engineering and human factors, and one with a background in nursing and human resource development). Composition of the local expert team differed per hospital, but generally consisted of one or two surgeons, one anesthesiologist, one or two OT-nurses, and one OT-manager. In two hospitals, a quality expert was part of the local expert team.

#### Steps in each iteration

**Fig. 1:**
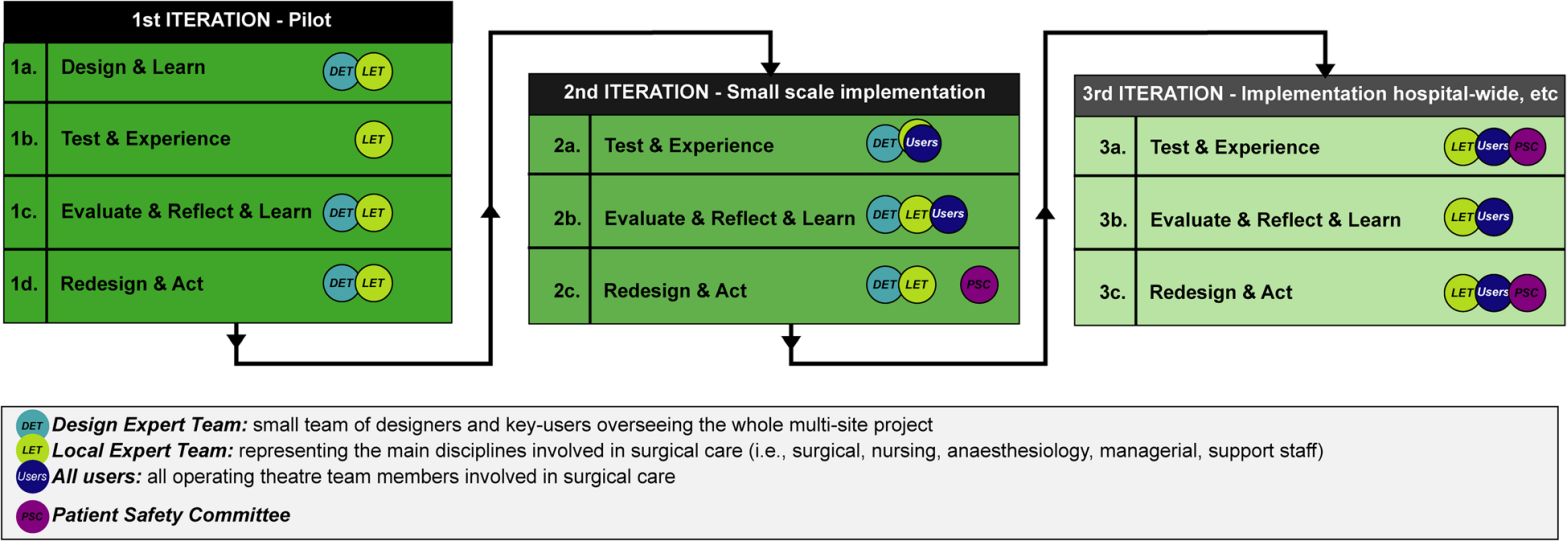
Adaptive Design model with its iterations and participants

The Adaptive Design (AD) model is visualized in Fig. 1.

- DESIGN & LEARN. The hospital’s local expert team functions as a steering group to provide the necessary information on the local context to adapt the prototype (if necessary). The design expert team supports the local expert team.
- TEST & EXPERIENCE. The prototype is tested by a small group of users to obtain feedback on content and usability and possible changes required in clinical processes. Feedback is self-reported on predesigned forms.
- EVALUATE, REFLECT & LEARN. The design expert team analyses the registered data and presents formal progress reports to the local expert team and all team members. Both teams look for additional information that requires revision of the basic criteria.
- REDESIGN & ACT. The design and local expert team discuss suggestions to redesign the prototype to ensure the best possible introduction and defines evaluation criteria for the TEST & EXPERIENCE step in the second iteration.

To ensure structural evaluation of the procedure following the second iteration, a patient safety committee is involved in the decision-making process and replaces the design expert team.

## Methods

In this multiple-site study we used Participatory Action Research (PAR) to explore the application of the Adaptive Design model with the design and implementation process of TOP*plus* in OTs of 10 Dutch hospitals (university, teaching and general hospitals). PAR was used as a research approach as it provides the opportunity for researchers to: “*… .. engage with participants as collaborators who can inform project design, propose methods, facilitate some of the project activities, and importantly review and evaluate the process as a whole” page 12* [24]. PAR improves implementation of innovations in iterative cycles, and at the same time gathers justifiable empirical data to create a scientific body of knowledge [25].

Using PAR, the researcher has a dual role as a researcher and as participant. At the (three) university hospital locations, the researchers (CD/LW) were actively involved as project leaders and thereby attained a better understanding of the problems encountered and professionals involved. In the other hospitals participating in the TOP*plus* study, the researchers’ roles were limited to researcher and project adviser.

Hospitals were not pre-selected, but joined the TOP*plus* study voluntarily. A selection was made to include different types of hospitals representing the three main types of hospitals in the Netherlands; University (U), Teaching (T) and General hospitals (G). As hospitals were about to implement new guidelines and protocols in OT and heard about the study, more hospitals joined. Ten hospitals were included in the TOP*plus* study: three university hospital locations (U), three affiliated teaching hospitals (T) and four general hospitals (G). The main reasons for participation were the integrated approach used in the TOP*plus* study and the scientific basis. The university hospital locations were regarded as separate hospitals since they differed in size, patient population, and type of care provided, but also in autonomy in policies, working procedures and budget.

**Fig. 2:**
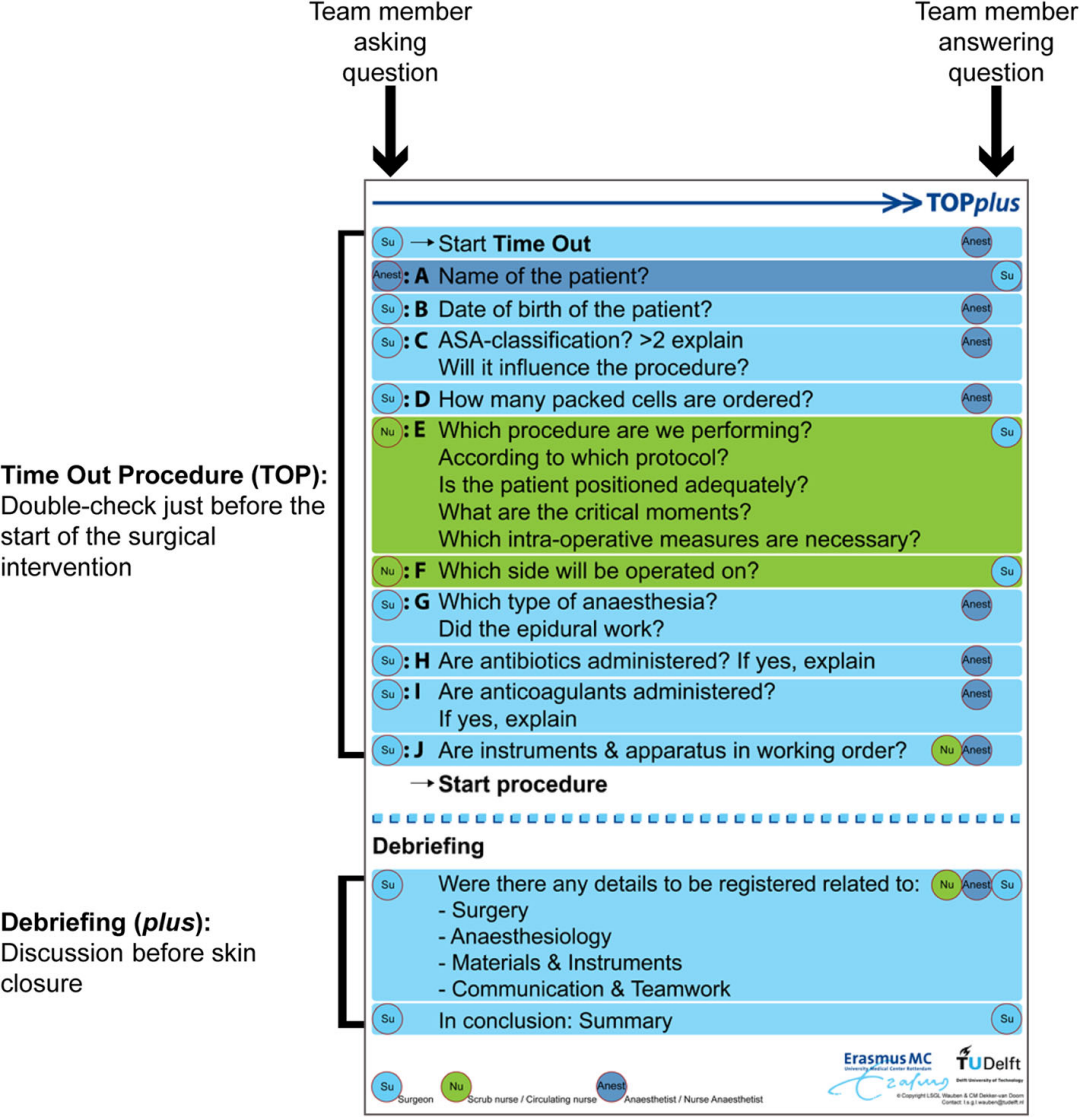
Basic TOP*plus* poster (prototype)

A basic TOP*plus* poster (the prototype) was developed to support team members in performing TOP*plus* in OT (see Fig. 2). The poster showed the questions and indicated the team member asking or answering questions: acting as a memory board for the OT team.

To inform all OT team members, a presentation was given providing background information on patient safety issues, the TOP*plus* procedure, and the project itself. All users also received written information about TOP*plus* by mail and e-mail. In some hospitals, the researchers also met with team members on the work floor.

**Fig. 3:**
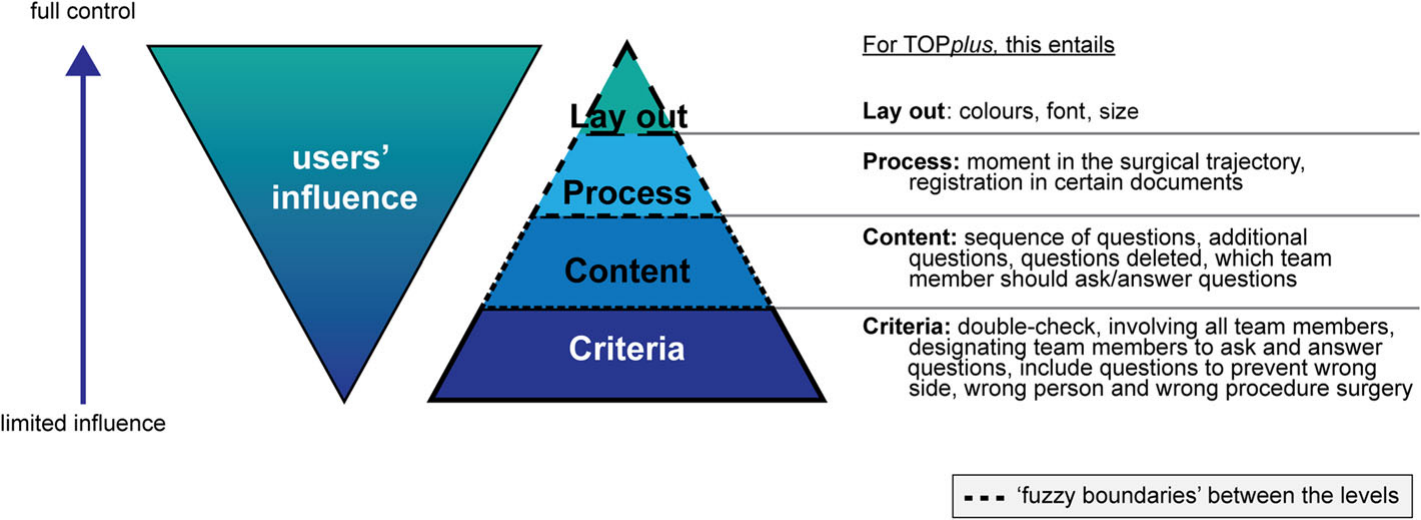
Four levels of end-user ‘s influence

To test actual usage and usability of the prototype, four levels of end-user influence were defined by the design expert team (see Fig. 3):

1. Criteria: items added because of new or adapted external guidelines, regulations or local needs;
2. Content: the sequence of questions, questions added, deleted, or rephrased, the team member designated to ask/answer the questions, and items discussed in the Debriefing;
3. Process: changes in the surgical care process and its effect on documents or systems;
4. Layout: colours, font or size of the poster.

The iterative character of Adaptive Design implies that with every iteration the team reflects on the effect of the proposed changes related to that specific level, e.g. does it violate the criteria, or does it affect the surgical care process [26].

The level of criteria, comprised items that are mandatory in the time out procedure to guarantee patient safety. These items were in line with national and international rules and regulations set by professional organisations, such as the World Health Organisation, the Joint Commission or by scientific professional associations [27, 28]. As TOP*plus* was a team intervention, it was also decided that all professional team members directly involved in the surgical intervention should be present. Prior to each iteration, developments at national and international level were checked to see if the criteria needed adaptation.

The content level consisted of questions to exchange critical information about the patient and the surgical intervention. Designated team members would ask or answer questions. With each iteration, team members were invited to give feedback and add, delete or rephrase questions, or change the designated team member asking or answering questions.

At the process level, the design and local expert teams decided at which moment in the surgical intervention the time out procedure and debriefing should be carried out. Again, with each iteration team members could propose changes.

At the layout level with the start of the project, the design expert team determined the layout of the posters and the colours used. Colours, structure and font were functional: the colours represented the different professional disciplines, the structure supported the procedure (the logical sequence of the items discussed) and the font should be easy to read from a distance. Following each iteration, users were free to change the layout. All changes were based on consensus after discussing the analysed data from the feedback, with the local expert team.

### Data collection and analysis

Applying the Adaptive Design model to TOP*plus* resulted in three iterations (Fig. 1). Preceding the first iteration (the start-up), the design expert team outlined a prototype for TOP*plus* (using expert opinion and relevant literature). Based on the information gathered, it was decided which items should be included in the procedure (see Fig. 2) [28, 29]. For the design of the prototype, see LSGL Wauben, CM Dekker-van Doorn, J Klein, JF Lange and RH Goossens [16]. To improve team communication and teamwork, requirements to emphasize the team character of TOP*plus* were established.

Data collection was carried out during four phases: 1) start-up: information sharing by presentations during meetings, and by mail and email, 2) experimenting with TOP*plus* during 100 surgical interventions (pilot, first iteration), 2) implementation on a small scale, including one or two surgical disciplines (second iteration), 4) hospital-wide implementation of TOP*plus* by all surgical disciplines (third iteration, etc).

During the iterations, data were gathered on usability and actual usage of TOP*plus* according to the four levels of end-users’ influence: the criteria, the content, the process and the layout of the PSP (see Fig. 3).

#### Adaptations

To measure all *adaptations* in the TOP*plus* procedure and in the surgical process during the iterations, (paper) evaluation forms were developed. In the first iteration (testing the prototype in 100 surgical interventions) detailed information was gathered on the alterations to adapt TOP*plus* to the local context. Data included:

- Date and the name of the surgical intervention;
- Content of TOP*plus*: were all questions asked and answered by the designated team member conform the poster, or by one of the other team members;
- Presence and active participation of each team member, if not why;
- Duration of both TOP and Debriefing;
- The moment (step) in the pre-operative process the TOP and Debriefing were performed.

Based on the results of the first iterations, data registration was reduced for subsequent iterations, and included: the date and the name of the surgical intervention, if TOP*plus* was performed according to the redesigned procedure, if all team members participated and if not ‘why?’, the duration of both procedures, details (incidents) in the four categories and additional remarks and suggestions.

All data were self-reported and manually registered by one of the OT team members (in most hospitals by the nurse anaesthetist) during or directly following the surgical intervention. The researchers (CD/LW) gathered and analysed the data. To validate the data and initiate the discussion between team members, both the data and the analysis were presented to and discussed with the local expert teams and in most hospitals with all team members in an evaluation meeting, following each iteration. The number of feedback moments, depended on the number of iterations needed for adaptation and full implementation hospital-wide. Again, all team members were invited to provide feedback. To inform all professionals involved in the design and implementation process, formal progress reports presenting all data and the analysis were distributed in hard copy and/or emailed to all professionals. Possible adaptations that would improve the procedure were discussed and, if consensus was reached by the majority of team members, implemented. Adaptations related to a specific discipline, e.g. Ear Nose Throat (ENT) surgery or ophthalmology, were discussed with that discipline and the involved OT team members. The adaptations were then tested with those teams in a new iteration.

Following consensus, poster adaptions would be made and implemented immediately. Since one of the researchers (LW) redesigned the posters to adapt the procedure as a result of the team discussion in the implementation process, all alterations to TOP*plus* and inclusion of more disciplines were carefully monitored.

#### Adoption

To measure *adoption* of TOP*plus* by healthcare professionals, data were gathered to determine if TOP*plus* was implemented hospital-wide in all OTs for clinical and ambulatory surgical care, and according to the protocol with all team members present and actively participating and not as a tick box exercise. In each hospital, the following data were gathered:

- Adoption of TOP*plus* by all surgical disciplines;
- Presence of the whole OT team during both TOP and Debriefing;
- Time for implementation hospital-wide, which included the initial start-up, the pilot, implementation with one or two disciplines, or implementation including all disciplines at once.

These data were also self-reported and manually registered by the same OT team member using the same evaluation forms as for collecting data on the adaptations.

## Results

In this study, the results of the ten hospitals are presented (U1–3, T1–3, G1–4). Almost all hospitals adapted TOP*plus* at content, process and layout level, but little was changed at the level of criteria. Adaptations are described at each level of influence; the criteria, the content, the process and the layout level of TOP*plus*. As far as adoption is concerned, all hospitals implemented TOP*plus* in clinical and ambulatory OTs with all elective surgical procedures. TOP*plus* was not always performed with all emergency surgical procedures. The presence of the whole team was high with the TOP, but fairly low with the Debriefing.

### Adaptation of TOP*plus*

#### Adaptations at the criteria level

In the first iteration (experimenting with TOP*plus* during 100 surgical interventions), all team members accepted the criteria without deleting or adding items. In the second iteration, one general hospital (G4) decided to change the TOP format. Rather than asking each other questions, the surgeon and anaesthesiologist informed the other team members about the patient, the surgical intervention and the anaesthetic intervention. Other team members were invited to cross-check and ask for additional information, thus keeping the team dialogue intact. At the three university hospitals (U1, U2, U3), the criterion ‘with all team members present’ was adapted by adding that the resident performing the surgical intervention could represent the staff surgeon provided he or she was actively involved in the surgical procedure.

#### Adaptations at the content level

Alterations to the content of TOP*plus* following the first iteration were limited to the TOP (see Table 2). Some questions were irrelevant for small surgical interventions and were deleted. For ambulatory care, for instance the question ‘Did the epidural work?’ was removed, as this kind of anaesthetic procedure is never used in ambulatory care. Some questions were added because of the complexity of the surgical intervention (e.g. ‘Are co-practitioners informed?’), the large number of people present in OT (e.g. ‘Does everyone know each other?’), or because the patients were participating in a research project (e.g. ‘Is this a study patient?’).

**Table 2:** Adaptations at Content Level

Questions		Start-up	Iteration 1	Iteration 2
**Questions reordered**	**TOP**	U1, U3, G4	U1, T1, T2, T3, G3	T2, T3, G4
	**DEB**	–	G3	–
**Questions added**	**TOP**	U1*, U3*, T2*, T3, G1, G3, G4	U1*, U3, T1*, T2*, T3*, G1*, G3	U3, T2*
	**DEB**	U1, U3, T2	U1*, T1, T3*	T2, T3, G4*
**Questions deleted**	**TOP**	U1*, U2*, U3*	U1*, U2*, T1*, T2, T3, G1, G2*, G3*, G4	G4*
	**DEB**	–	U2, G3	G4*
**Questions rephrased**	**TOP**	U1*, U3*, T2*, T3*, G3, G4	U2*, T1*, T2*, T3*, G2*	U3, T2*, G4*
	**DEB**	–	T1	T2*, G4*
**Procedure**				
**Designated team member asking question**	**TOP**	U3*	U1*, U3	U3*, T2*, G4
	**DEB**	U3	U1	T2
**Designated team member answering question**	**TOP**	U3*	–	U3, T2, T3
	**DEB**	U3*	–	T2, T3

TOP: Time Out Procedure

DEB: Debriefing

* Hospitals making more than one adaptation

Following the second iteration, to improve patient handover from OT to recovery, three hospitals (T2, T3, G4) added additional questions to the Debriefing about post-operative care addressing ‘postoperative orders’ or ‘additional diagnostic lab work’. Because nurses are always present, three hospitals decided that the nurse anaesthetist (T2, G4) or circulating nurse (U3) would ask all questions.

#### Adaptations at the process level

Adaptations at the process level were primarily related to the moment in the surgical process the TOP was performed. Some team members stated that performing the TOP just before incision was too late. Errors like wrong patient or wrong site should be detected and corrected before induction. To perform the TOP before induction appeared to be difficult, as it interfered with existing surgical routines, such as early-morning patient handover, which requires the presence of all surgeons and residents. To solve this problem, the three university hospital locations decided that the residents could replace the surgeon (U1, U2, U3). Six hospitals (U2, U3, G1, G2, G3, G4) adapted the pre-operative process by adding a pre-anaesthesia check. Four hospitals (U1, T1, T2, T3) decided to perform the TOP before induction and introduced a second comprehensive TOP just before skin incision. In the children’s university hospital (U1) the team decided to perform the TOP without the patient being present because it would be too stressful for children.

All adaptations were regarded as appropriate solutions making TOP*plus* effective within their specific local hospital context improving patient safety.

Experimenting with 100 surgical interventions in the first iteration resulted in additional comprehensive TOPs for small surgical interventions. Like TOPs for small interventions that required only local or regional anaesthesia, mostly in ambulatory care (G3) of for small interventions in e.g. ENT surgery and ophthalmology (U1, U2, T1, T2, G1, G2) in acute clinical care.

In the second iteration, hospitals U1 and U3 introduced a TOP to structure patient handover between the clinical ward and OT. ENT specialists in hospital U1 designed and implemented another initiative that introduced two new TOPs provided the OT team did not change: 1) a ‘TOP-5’, to discuss five small consecutive ENT-interventions, and 2) a comprehensive TOP, in which only the correct intervention, the correct surgical site and the correct patient were confirmed again just before each intervention.

#### Adaptations at the layout level

Only four hospitals made changes at the layout level (U1, U2, T2, T3). Over time, the TOP*plus* layout became the standard in most participating hospitals and as a daily routine procedure and turned into a verb “toppen”. Following the first iteration, U1 combined the pre-anaesthesia TOP and the pre-incision TOP, performed in two different rooms, into one poster. For each room on the TOP*plus* poster the TOP to be performed in the other room was de-emphasised (smaller and grey font). Hospital U2 combined the basic TOP*plus* and the adapted TOP*plus* for small interventions into one poster. Following the second iteration, T2 and T3 adapted the layout and colours of the poster to correspond with the hospital’s corporate house-style.

### Adoption of TOP*plus*

#### Adoption by all surgical disciplines

Five hospitals implemented TOP*plus* immediately in all OTs and with every surgical procedure (U2, U3, T1, G3, G4), and five hospitals started with the surgical procedures of one surgical discipline (U1, T2, T3, G1, G2). In all hospitals, at least two different TOP*plus* procedures were implemented in all OTs, one for complex, clinical surgical procedures and one for less complex, mostly ambulatory, surgical procedures. Differences in items to discuss in the TOP resulted in different TOP*plus* posters to support adoption. In two hospitals (G2, G4) extra posters were designed for a “pre-TOP”, a TOP to be performed before induction, and in four hospitals (G1–4) extra posters for time out procedures and debriefings for different disciplines and different interventions were designed.

### Presence of the whole team

Initial user participation varied between hospitals from around 80% (U1, U2, U3, T2, T3) to around 50% (G1, G3, G4, T1). In one hospital (G2), participation was only 40%. In some hospitals, the surgeon would not be present for the TOP, in most cases, because of the patient handover early in the morning with all staff members and residents. With some disciplines, resistance was high and it was only over time, that surgeons would see the advantage of such a procedure and cooperate.

In most hospitals, the presence of the anaesthesiologist at the Debriefing was low. Sometimes because of an emergency patient admitted for surgery, but mostly, because (Dutch) hospitals use a ‘two-table system’, meaning that the anaesthesiologist works in two OTs. At the end of the surgical intervention, just before skin closure and the start of the Debriefing, the anaesthesiologist leaves OT to prepare the next patient for surgery. The nurse anaesthetist takes over and is responsible for recovery of the patient.

Ultimately almost all team members in each of the hospitals were actively involved in the design and implementation process and adopted TOP*plus* as a daily routine procedure. The fact that in some hospitals the nurse anaesthetist would ask all questions did not affect the team aspect of the discussion. All team members still had to be present in order to answer questions and provide specific information individually.

### Time for implementation hospital wide

**Fig. 4:**
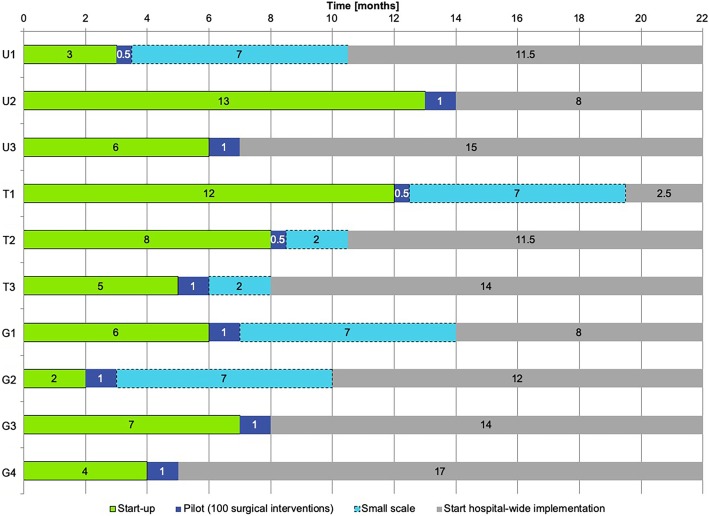
Time for implementation of each of the four phases

The time to include all surgical disciplines varied between hospitals. Figure 4 presents the time hospitals needed for each of the four phases. In all hospitals, TOP*plus* was introduced with all elective clinical surgical procedures.

The largest differences were found in the period preceding the pilot, the start-up phase. In the initial planning of TOP*plus*, one to two months were reserved for the start-up phase. This phase merely included meetings with the local expert team to discuss the TOP*plus* study and oral presentations for all professionals directly involved in surgical care and information sharing by mail or email. Two hospitals (U2, T1), spent a significant amount of time discussing the project getting the means and support. Only one hospital (G2) stayed within the initially planned time of two months. Although, the three hospitals (U2, T1, T2) that spent most time in the start-up phase were large hospitals, two of the smaller hospitals (G1, G3)) needed a lot of time as well.

The duration of the first iteration was the same for all hospitals; teams tested the prototype with 100 surgical interventions and data were gathered within two weeks. The next phase, implementation on a small scale and hospital-wide implementation, varied per hospital. Some hospitals decided to involve all disciplines (and thus all OTs) at once. Others decided to include different disciplines successively over time. Most hospitals completed the iterations to implement TOP*plus* hospital-wide within one year. From start-up till the start of hospital-wide implementation, the university and teaching hospitals required an average of 10.5 months and 12.7 months respectively, the general hospitals an average of 9.3 months. Most hospitals needed at least three iterations to adapt TOP*plus* to the local context, the surgical disciplines or the type of care provided (clinical or ambulatory). Most discussed were the following three topics: 1) whether to perform the TOP before or after induction, 2) the necessity of having all team members present for both the TOP and the Debriefing, and 3) whether and to what extent TOP*plus* could be adapted to meet local needs.

### Effects at interdepartmental level

The implementation of TOP*plus* in OT also initiated discussions with other medical disciplines. Over the course of the study, similar procedures were designed for complex interventions with other medical disciplines, such as intervention cardiology, intervention radiology, oncology (chemotherapy) and obstetrics. Implementation of TOP*plus* also initiated the design and implementation of a checklist covering all of the critical steps in the surgical care path from admission to hospital discharge (U1, U2, U3).

## Discussion

Combining Participatory Design and Experiential Learning principles into the Adaptive Design model appeared to be successful for adaptation and adoption of TOP*plus* by all professional disciplines and all professionals. The results showed that each hospital adapted TOP*plus* to its own needs and specific local context. Adaptations of both TOP and Debriefing were primarily made at the content and process levels, following the first iteration of 100 interventions. The level of criteria, however, remained fundamentally unaltered, thereby keeping essential features for the effectiveness of TOP*plus* intact. All hospitals implemented TOP*plus* with all surgical disciplines in all OTs.

Differences in the hospital-wide implementation process were mostly by choice. Some hospitals decided to start implementation with all surgical disciplines, directly after the pilot, others took a more stepwise approach and included more disciplines over time. The time preceding the pilot mainly differed but was well spent. The ability to influence every step in the process from the first step on, created time for discussion and reduced resistance among professionals. They valued the possibility to adapt the procedure step by step, which made it feel like their own initiative. When professionals are allowed to experiment and results are visible, chances that the new procedure is perceived as practical and useful increase [15, 30, 31]. The information gathered during iterations, supported shared decision-making about appropriate adaptations, which were then implemented step-wise and re-evaluated. To create ‘a fertile ground for change’, it is important to put the professional in the lead, provide frequent structured feedback and actively involve all team members [30]. EF Taylor, RM Machta, DS Meyers, J Genevro and DN Peikes [4] used a similar approach and showed that co-design and a focus on local context, results in a better uptake of national recommendations.

Adoption was facilitated by experimenting and learning collectively as a team. The team learning process created openness among team members to discuss each other’s tasks and responsibilities. Active involvement of professionals enables teams to create a shared mental model and gain insight in tasks and goals [32, 33]. The learning cycles and the possibility to adapt the TOP to their own patient group or surgical intervention created a safe environment to experiment. This reduced resistance, increased motivation and acceptance, and supported the adoption process [15, 34, 35]. The possibility to adapt the TOP also reduced reluctancy to involve patients; by now active engagement of patients in the care process is fully accepted, however not always practiced.

In many cases, however, a top-down approach is used for the introduction of PSPs, where questions on what to change and how are decided by administrators and managers [36]. The WHO surgical safety checklist for instance, launched as the solution to decrease preventable complications and deaths [37], showed a positive effect on morbidity and mortality rates in nine different countries [18]. However, in a later study questions were raised about the mandatory and universal adoption of the checklist, as results showed no significant effects on a decrease in complications or a lower mortality rate [38]. We think that our Adaptive Design model using a bottom-up approach and engaging front-line staff in iterative cycles of design, test, experience & evaluate, and redesign supports actual usage of the procedures by all professionals.

In this study, rather than presenting TOP*plus* as the solution for a problem, we used a bottom-up approach to improve patient safety. Recent studies make a plea for similar qualitative research methodologies, such as Participatory Learning and Action Research (PLA) [39, 40]. Like Adaptive Design, PLA creates a structure to engage front-line staff in the design and implementation process, and improve patient care and generate reliable and justifiable data. Another study by L Jeffs, J McShane, V Flintoft, P White, A Indar, M Maione, AJ Lopez, S Bookey-Bassett and L Scavuzzo [41] using a similar approach by creating deliberate and structured learning activities, helped teams to achieve outcomes that were realistic and meaningful.

This study has some limitations. Voluntary participation and the supportive nature of the project might have influenced the results positively, but did not guarantee actual participation of all professionals. At some hospitals, resistance was met and only decreased by allowing time for an extra iteration to experiment and learn. However, this is exactly the aim of AD, it is not *“a simple linear process something ‘done’ to people”* but a strategy to encourage team members to decide what works best in their local context, based on evidence [31]. Another limitation was the dual role of the researchers, which challenged the researchers’ ability to remain objective. To prevent bias, team member checks were carried out by presenting the data and analysis and discuss the results with the local expert teams and in most hospitals with all team members. A third limitation is the lack of a control group and the absence of extrapolation to other hospitals. Research in safety culture, work climate and incident reporting shows a large variability between organizations, departments and professional disciplines [42, 43]. As safety culture influences professionals’ attitudes and behaviour, this makes it difficult to generalize the results to other groups or organizations. We explicitly used Participatory Action Research to explore if and how Adaptive Design could be effective, find out what works and what not and validate the method used. Future research should use a longitudinal or stepped wedge approach to measure results over time and include a multi-level analysis. This study focussed on what needed to be changed (the content of TOP*plus*, or when to perform TOP*plus*) to fit TOP*plus* to the local context and the needs of the professionals. In a future study we will evaluate the implementation process from the viewpoint of the users, the professionals (i.e. the rational for the changes, who initiated the changes, and the variations in participation).

## Conclusions

Although this study focussed on a specific patient safety practice, it shows that using a structured bottom-up implementation approach with a focus on learning (and thus engaging front-line staff) improves adaptation and adoption of a patient safety practice. Every hospital has its specific local circumstances that need to be taken into account in the process of implementation. The Adaptive Design model provides a structure to create that ‘fertile ground for change’ and decreases resistance for successful implementation.

## Data Availability

The datasets used and/or analysed during the current study are available from the corresponding author on reasonable request.

## Abbreviations

AD: Adaptive Design
EL: Experiential Learning
ENT: Ear Nose Throat
G: General hospital
OT: Operating Theatre
PAR: Participatory Action Research
PD: Participatory Design
PDCA: plan–do–check–act cycle
PLA: Participatory Learning and Action Research
PSP: Patient Safety Practices
U: University hospital
T: Teaching hospital
TOP*plus*: Time Out Procedure (TOP) and Debriefing (*plus*)
WHO: World Health Organisation
